# Migrant physicians’ entrance and advancement in the Swedish medical labour market: a cross-sectional study

**DOI:** 10.1186/s12960-019-0414-0

**Published:** 2019-10-15

**Authors:** Linda Sturesson, Magnus Öhlander, Gunnar H. Nilsson, Per J. Palmgren, Terese Stenfors

**Affiliations:** 1 Department of Learning, Informatics, Management and Ethics, Karolinska Institutet, 171 77 Stockholm, Sweden; 2 Department of Ethnology, History of Religions and Gender Studies, Stockholm University, 106 91 Stockholm, Sweden; 3 Department of Neurobiology, Care Sciences and Society, Karolinska Institutet, 171 77 Stockholm, Sweden

## Abstract

**Background:**

Worldwide, physicians are migrating to new countries and want to practise their profession. However, they may experience difficulties doing so. To optimise and accelerate their entrance into and advancement within the Swedish healthcare system, there is an urgent need to explore how they are currently doing so, as their competences should be put to use without any unnecessary delay. The aim of the study was to explore how migrant physicians with a medical degree from outside EU/EEA enter and advance within the medical labour market in Sweden and to identify perceived barriers and facilitating aspects in the process. The empirical findings are discussed in light of Bourdieu’s concept symbolic capital as adapted in the Swedish medical field.

**Methods:**

A cross-sectional study with a self-administrated questionnaire was disseminated. A sample of 498 migrant physicians were identified. Descriptive statistical analysis and qualitative thematic analysis were used to analyse the data.

**Results:**

The response rate was 57% (*n* = 283). Respondents mainly found their first positions via spontaneous job applications, during internships, while participating in an educational intervention or via personal contacts. Perceived barriers to entering and advancing within the medical field in Sweden were mainly related to having a medical degree from and/or originating from another country, which could in turn represent discrimination and/or having one’s competence undervalued as a result. Facilitating aspects included having or developing contacts in Swedish healthcare and gaining proficiency or fluency in the Swedish language.

**Conclusions:**

When MPs find their first positions, the contacts they have developed appear to play a role**,** and when advancing in their positions, the active development of a variety of contacts seems to be beneficial. MPs experience a variety of barriers to entering and advancing within the field that could be related to discrimination. Many MPs perceived having their competences undervalued due to their origin or to being educated abroad. Based on the respondents’ experiences, our interpretation is that MPs as a group are hierarchically positioned lower in the Swedish medical field than physicians trained in the country. Facilitating aspects included educational interventions, having contacts and developing language skills. For optimal entry into the labour market, it is vitally important for MPs to learn the new language and obtain a job or internship in the field as soon as possible.

## Introduction

For migrant physicians (MP) with a medical degree from abroad who want to enter the medical labour market in their new country as practising physicians, different routes and interventions have been developed [1–8]. Empirical investigations on MPs often focus on their arrival and the early stages of recertification processes [1, 9], on educational interventions [1, 10] or on challenges, barriers and facilitating factors for workplace integration, i.e. when MPs have found employment [1, 9–14].

Less research exists on the process between an educational intervention and/or recertification and employment, although available findings indicate that MPs struggle to find employment [8, 15]. One barrier to entering the labour market for migrants is discrimination [8, 16–20], especially for those from non-western countries [17–20]. Highly skilled migrants are no exception [19, 21]. Some have experienced being undervalued at work despite their education [22]. This might also occur during the job-seeking process (cf. [23]), yet this issue has not been thoroughly explored. Other barriers to employment include a lack of knowledge about how to apply for work or how to act in a job interview, as well as language problems [19]. Also, as the recertification process takes time, many MPs are often older than peers educated in the new country [8, 19], and they might have families which can make it more difficult to study (cf. [8]). Empirical evidence also suggests that having children is a barrier as it reduces possibilities to move [19] to for example rural and remote areas where the absence of physicians are common, and MPs seem to prefer to live and work in urban areas [1, 24–26].

In Sweden, MPs trained outside the EU/EEA, as well as physicians trained in Sweden, have to apply for and complete a mandatory medical internship (MMI) and must pass an assessment before they can obtain a Swedish licence to practise. The number of MMI positions is, however, limited, which in turn results in longer waiting times [27]. Governmental reports suggest that MPs take longer than their Swedish-educated peers to obtain their Swedish licence to practise [28]. This may be because MPs lack the networks that might be needed to secure an MMI position [28].

From a societal and individual perspective, it is crucial that non-EU medical educated MPs’ competences can be put to use in the labour market without any unnecessary delay. To optimise and accelerate MPs’ entry into the Swedish healthcare system, there is an urgent need to explore how MPs currently enter into and advance within the system. Thus, the aim of this study is twofold: (1) to explore how MPs enter into and advance within the medical labour market in Sweden and (2) to identify perceived barriers and facilitating aspects for entrance into and advancement within the Swedish medical labour market.

One way to explore how MPs becomes a part of the Swedish medical workforce and advance within the medical labour market is to employ the concepts of *social field* and *symbolic capital* developed by Bourdieu [29, 30]. Simply put, a social field can be defined as a limited system of relations between social positions (individuals) with shared interests. In this article, we will use the concept *medical field*. The medical field comprises different kinds of medical professionals, positions for whom are allocated based on negotiations about common interests, including characteristics of medical science, medical work or medical competence [31]. Different types of symbolic capital, which can be cultural, social or economic, position individuals hierarchically in a given field. When capital is recognised in a specific field, it becomes symbolic capital, which thereafter defines a person’s position and possibilities within that field. Having a recognised education and degree, as well as requisite skills and knowledge, can be understood as conferring symbolic capital (cultural). Symbolic capital (social) can also manifest as a valuable and useful network within a profession. To be counted as symbolic capital, however, such a network should potentially lead to a gain—for example, employment (cf. [32]). Blain et al. (2016) suggested that symbolic resources influence opportunities for MPs to work in a medical field [33]. Having social capital in the form of a useful social network may thus facilitate employment.

The medical field can be understood as transnational, as it extends beyond national borders, and physicians can practise their profession in different countries [34]. However, a smooth transition from one country’s medical field to that of another country cannot be guaranteed. In the process of migration, a professional’s capital has to be recognised in the new country’s specific medical field. Depending on where a medical degree is acquired, its accompanying cultural and social capital might not be valued equally. For example, when physicians with a medical degree from outside the EU/EEA want to enter the medical field in Sweden as independently practising physicians, their medical degrees must be formally acknowledged before they can acquire a Swedish medical licence. Thus, MPs’ symbolic capital can, based on jurisdiction, be seen as being devalued when transferred to a new country’s medical field (cf. [23]). Accordingly, MPs must have their capital recognised and create new capital, which can be termed *migration-specific capital* [35]. Migrants also actively develop a ‘mechanism of validation for their cultural capital’ in the bargaining process when trying to enter the field [35]. One validation route is the institutionalised Complementary Program for Physicians (CPP). Via the CPP, MPs are expected to develop the country-specific competence needed to obtain a Swedish medical licence to practise. In this new country, with its dominant culture, MPs must further develop their symbolic capital for it to be of any real value (cf. [35]). The cultural capital (e.g. education, skills and knowledge) developed in another country’s medical field might represent a barrier if it is valued less than that of the new country.

## Methods

For our research, we applied a cross-sectional study design. A questionnaire was distributed to a sample of MPs in Sweden who possessed a medical degree from outside the EU/EEA or Switzerland.

### Context

Physicians with a medical degree from outside the EU/EEA or Switzerland can follow three routes to obtaining a Swedish licence to practise: (1) take a proficiency test and pass a course in Swedish laws and regulations, after which they must find and complete 6 months of clinical training; (2) participate in the CPP for 10 months; and/or (3) participate in Swedish medical education.

Routes 1, 2 and 3 can be seen as one phase in the licencing process. Until June 2016, all three routes had to be followed by another, stand-alone, phase: the 18–21-month MMI, which is assessed by a test. After July 2016, the MMI are however only applicable to those beginning route 2 or 3 after July 2016.

Some MPs change routes. For example, an MP may begin route 2 (CPP) but then take the proficiency test (route 1) to accelerate the licencing process. Before the CPP and the MMI, and before obtaining the Swedish licence to practise, MPs (as well as physicians educated in Sweden) may work as junior doctors, which is a restricted position before being licenced.

### Participants and sampling

**Fig. 1:**
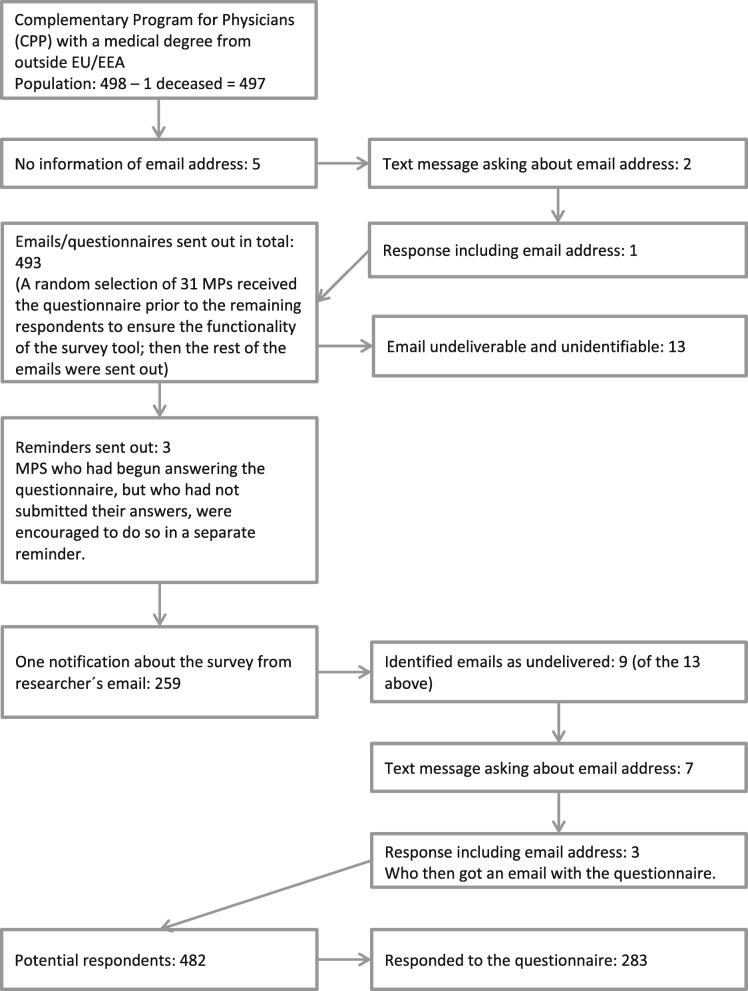
Sampling frame

Potential participants for this study included MPs with a medical degree from outside the EU/EEA or Switzerland who enrolled in the CPP (route 2) during the 2009–2017 admission years—in total, 498 MPs. Route 2 was selected due to access to demographic data. (Unfortunately, one was deceased) CPP/route 2 potential participants were identified via registry data provided by the universities giving the programme. The sampling strategy was non-probabilistic and had the potential to include the whole population of route 2 participants, giving them an equal opportunity to participate in the study so long as they had updated their contact details in the registry. In Fig. 1, a detailed sampling frame is presented.

Participation was voluntary, and written informed consent was obtained from the participants. All collected data were anonymised to maintain the integrity of the individual respondents, and the data were handled and stored in accordance with the tenets of the World Medical Association Declaration of Helsinki (2008). The Regional Ethical Review Board in Stockholm, Sweden, approved the study (2017/1717–31/5).

### Data collection

A self-administered questionnaire was developed to fit our aim. The questionnaire was developed following a seven-step process outlined by Artino et al. [36], as presented in Table 1.

**Table 1:** Development of the questionnaire

Step		Procedure
1	Literature review	When conducting the literature review, little empirical knowledge was identified regarding MPs’ pathways into the labour market in Sweden.
2	Interviewing	In a process parallel to the literature review, 24 participants from the CPP were interviewed at the beginning of the programme and after completing it (*n* = 16). Informal interviews with experts, such as recruitment staff, a physicians’ trade union, researchers and responsible persons from the CPP, were also conducted (*n* = 7).
3	Synthesising	Themes for further investigation were developed based on the literature review and interviews.
4	Item development	The themes were described, and questions as well as response scales were developed and discussed continuously [37–39].
5	Feedback	Questions were presented and critically discussed in different forums (e.g. research seminars) to obtain feedback on the themes, questions and wordings, as well as to streamline the questionnaire, fill any gaps and reduce redundant questions.
6	Cognitive interviews (CIs)	The constructed questions and response scales were discussed during individual interviews with six physicians, who were migrants *not* educated in Sweden (except one). After the cognitive interviews, the questionnaire was further improved regarding wording, formulation and response alternatives.
7	Pilot test	I) The questionnaire was constructed digitally with a survey tool and sent out for pretest to 23 individuals. In this pretest, 16 respondents provided feedback on the logic in the questionnaire, as well as on the questions and response alternatives. The respondents consisted of researchers, people responsible for or working with the CPP, and others with relevant expertise. The questionnaire was thereafter further improved. II) The questionnaire was sent out for a pilot test to 24 physicians working in Sweden who were at different stages in their careers; however, this did not include a sample from the MPs selected for participation in the study. Some of the respondents in the pilot study had their medical education in Sweden. Fifteen of the 24 responded in the pilot. In the pilot, the respondents answered the questionnaire, but they also had the opportunity to provide feedback on the questionnaire, questions and wording. The results and comments from the pilot were discussed during two research meetings with different participants, and the questionnaire was refined twice more before use.

The final questionnaire comprised the following sections: participants’ backgrounds and previous experiences, entrance into the labour market, interventions and the MMI. The questionnaire was composed in Swedish and included open-ended questions without character limitations and close-ended questions with binary, nominal or ordinal response options. Depending on the participants’ responses, different follow-up questions were used. Hence, no respondent received the exact same set of questions. The questionnaire was distributed digitally and answered during February and March 2019. The sampling frame in Fig. 1 presents the process, the number of reminders and the number of potential and actual respondents.

### Data analysis

The completed questionnaires were exported to Excel. Descriptive statistics, which formed the backbone of the statistical analysis, were conducted on categorical data, which were subsequently presented as frequencies and percentages. The responses to closed- and open-ended questions were analysed to complement the quantitative results and deepen knowledge on the topic (cf. [40–42]). Thematic content analysis was used in this process [43]. The responses were read through for familiarisation, and significant sentences and/or keywords were identified and noted; then, central concepts were deductively grouped into the identified preset themes of *used strategies*, *experiences of discrimination* and *valuing of competence* (Table 1) (cf. [44]). The text answers were also analysed inductively to identify other themes related to barriers and facilitative aspects in the respondents’ answers (cf. [42, 44]). The findings were iteratively discussed between the authors to enhance credibility. Quotations were translated into English and edited to increase readability and protect confidentiality, without sacrificing the content or its meanings.

## Results

We present the results as follows: ‘Response and respondents’ demographics’, ‘How MPs enter into and advance within the medical labour market in Sweden’, ‘Experienced barriers’ and ‘Facilitating aspects’.

### Response and respondents’ demographics

The questionnaire had a 57% response rate (*n* = 283) (Table 2). The internal non-response bias was less than 10% on each question. The number of respondents (*n*) varied since the physicians were at different stages in their careers, and thus, not every question was relevant for everyone. Comparing all the participants who had begun the CPP, the distribution was equal regarding gender, year of birth and frequency of obtained Swedish medical licences. The respondents had resided in Sweden for an average of 10.3 years at the time the questionnaire was administered (*n* = 274). Respondents’ demographics are presented in Table 3.

**Table 2:** Distribution of eligible respondents and respondents by gender, birth year and possession of medical licence

	Category	Respondents (*n* = 283 (%))	Number of participants in the Complementary Program for Physicians (CPP) with a medical degree from outside the EU/EEA (*n* = 498 (%))
Gender*	Females	153 (54)	264 (53)
	Males	130 (46)	234 (47)
Year of birth*	< 1970	29 (10)	67 (13)
	1971–1980	104 (38)	183 (37)
	1981 >	146 (52)	248 (50)
Mean age		39 years	40 years
Obtained Swedish medical licence*	Yes	112 (40)	197 (40)
	No	171 (60)	301 (60)

*Registry data

**Table 3:** Distribution of respondents’ demographics

	Category	Frequency (%)	Comment
Region of origin	Asia	132 (55)	The respondents (*n* = 241) represented 43 countries (including Sweden). Some had grown up in more than one region.
	Europe	64 (27)	
	Africa	32 (13)	
	Latin America and the Caribbean	21 (9)	
	North America	2 (1)	
Region of medical education (*n* = 263)	Asia	131 (49)	The number of countries for education was 52 (*n* = 269). Some participants mentioned more than one country.
	Europe	88 (33)	
	Africa	28 (10)	
	Latin America and the Caribbean	21 (8)	
Year of graduation from medical education (*n* = 270)	2000 or before	50 (19)	
	2001–2010	142 (53)	
	2011 or later	78 (29)	
Years worked as a physician before migrating to Sweden (*n* = 279)	None	53 (19)	
	Less than 1 year	41 (15)	
	1–2 years	88 (32)	
	3–5 years	68 (24)	
	6–10 years	19 (7)	
	More than 10 years	10 (45)	

Of the respondents, 61% had worked in Swedish healthcare previous to enrolling in the CPP, on average for 20 months. Most had worked as assistant nurses, but some also held jobs as medical assistants and care assistants. A few had worked as junior doctors (a restricted position available before being licenced) or administrators, and some had worked as nurses, interpreters, in research or teachers of related healthcare-related subjects. Also, some respondents had taken internships and/or had been auscultating. It was common to have held several different positions.

When answering the questionnaire, 88% of the respondents currently held a position as a physician but were in different stages of their careers, as they had also started the CPP in different years (Table 4).

**Table 4:** Distribution of respondents (*n* = 249) with a position as a physician by type of position

Position	Frequency (%)
Junior doctor position (before the mandatory medical internship)	64 (26)
Mandatory medical internship position	65 (26)
Junior doctor position (after the mandatory medical internship and without having obtained the Swedish medical licence)	7 (3)
Junior doctor position (after the mandatory medical internship and having obtained the Swedish medical licence)	11 (4)
Specialty training position	94 (38)
Specialist position or senior doctor position	8 (3)

Of the respondents, 12% (34, *n* = 283) did not hold a position as a physician in Sweden when answering the questionnaire, but some held other jobs in the healthcare field or worked as physicians outside of Sweden. A few were unemployed, on parental leave or travelling.

### How MPs enter into and advance within the medical labour market in Sweden

Of the respondents who had applied for a position as a physician after the CPP (*n* = 265, 249 working as physicians, and 16 not working as physicians), 42% had applied for 1–5 positions, 9% for 6–10 positions, 8% for 11–20 positions and 8% for 21–50 positions. Five percent of the respondents had applied for 51 or more positions. Of the respondents, 29% stated that they did not need to apply for work. In Table 5, the different channels used by respondents to find their first job as a physician are presented.

**Table 5:** Distribution of respondents (*n* = 257) who received their first position as a physician after the CPP

Type of information (%, of *n*, % of answers in total *n* = 327)	Sub-types	Frequency (% of *n*, % of answers in total *n* = 327)
Contact with employer (68, 54)	Spontaneous job applications	73 (28, 22)
	During internship CPP (route 2)	63 (25, 19)
	Work previous to the CPP	24 (9, 7)
	During internship during route 1 (possible for 25 respondents)	15 (6, 5)
Personal contacts (23, 30)	Via friends	35 (14, 11)
	With study peers from the CPP	22 (9, 7)
	Via family or relatives	10 (4, 3)
	Via teachers in the CPP	6 (2, 2)
	Via mentor, coach or sponsor	3 (1, < 1)
Labour office (6, 8)		21 (8, 6)
Job advertisements (13, 17)	Country Council webpage	22 (9, 7)
	Employer’s webpage	17 (7, 5)
	Physicians Union Paper	4 (2, 1)
Social network contacts (2, 3)	LinkedIn	3 (1, < 1)
	Chat groups with other MPs	2 (< 1, < 1)
	Recruitment fair	1 (< 1, < 1)
	Staffing agencies	1 (< 1, < 1)

CPP denotes Complementary Program for Physicians with a medical degree from outside the EU/EEA or Switzerland

In most cases, the first job for the respondents (*n* = 263) after the CPP was as a junior doctor (88%, *n* = 232), while 3% (*n* = 9) worked as medical assistants. The ideal position after having completed route 1 or route 2 (the CPP) was, however, the MMI, which was the first position for 3% (*n* = 7) of the respondents. The average time to begin the MMI (waiting time) was 16 months (*n* = 179) and, in the largest urban area, 20.4 months (*n* = 29). One quarter (26%) of the 248 respondents who held a position as a physician (junior doctor, medical assistant or had an MMI) as their first position after the CPP got their first job offer during the CPP. One half of these respondents got their first job offer 0–3 months after completing the CPP (route 2) or route 1 (some had changed routes). After 4–6 months, 12% had received and accepted a job after having completed the CPP or route 1; for 7%, it took 7 months or more. For a few respondents, their first job was as an assistant nurse, but they had also held other jobs.

### Experienced barriers

We found several barriers to entering and advancing within the medical field in Sweden (Table 6). The themes of *having another ethnicity and/or religion or other belief*, *having a different name*, *insufficient language skills*, *having the wrong age* and *having the wrong gender* are associated with discrimination in Sweden [45]. In total, 29% of the respondents perceived having been disfavoured based on grounds related to discrimination during their job-seeking processes. The distribution of their experiences was equal in terms of gender and total respondents (Table 2): 44 women (54%) and 38 men (46%). However, there were some gender differences with respect to different grounds for discrimination, as displayed in Table 6. The answers showed that perceptions of having been disfavoured were related to several factors—for example, ethnicity, religion and gender.

**Table 6:** Barriers to and facilitating aspects and strategies for entry into and advancement within the medical labour market in Sweden

− Barrier	+ Facilitating aspect and strategy
**Originates from another country and/or has a medical degree from abroad** Origin from another country was a perceived reason by 16% of the respondents (*n* = 283) for having one’s competence undervalued during the job-seeking process compared to physicians with a medical degree from Sweden. ‘*They have said it straight out in the interview without knowing or knowing what knowledge I have. On several occasions I heard prejudices about the country I was trained in*’*.* Others did not believe that their medical knowledge was enough, which was a perceived reason for having competence undervalued by 11% of the respondents (*n* = 283). One respondent wrote: “*I worked as a [names a specialty] for [XX] years, unfortunately my experience became ‘zero’*”. To have a medical degree from abroad was a perceived reason for 6% of the respondents for having their competence undervalued. ‘*Worked as a junior doctor and had been promised a mandatory medical internship; however, physicians who had never worked as junior doctors got their mandatory medical internship before me, probably since they were educated in Sweden/EU*’. ‘*There is many/almost everyone who does not trust foreign physicians*’. ‘*Got to hear from a medical director at a geriatric clinic where I applied for a job that we know nothing, can do nothing, and that we are burden on the Swedish system, and that they do not even want to meet a doctor like us*’.	**Not all respondents were born abroad, but may have moved during childhood and then returned** ‘**It has been an advantage being born and raised in Sweden and speaking Swedish fluently, in comparison to CPP peers**’. **Applying for Swedish citizenship** As a strategy to increase job opportunities, 11% of the respondents had applied for Swedish citizenship. **Having experience from another country** ‘*My experience from my home country helped me a lot*’.
**Having another ethnicity and/or religion or other belief** Having another ethnicity and/or having another religion or other belief is not the same thing, but can be connected in how people perceive each other. Of the respondents, 20% perceived that they had been disfavoured related to discrimination by having another ethnicity, and 7% perceived that they had been disfavoured related to discrimination by having another religion or other beliefs. ‘*Regarding Muslims and Arabs, they think we have a bad view on women*’. ‘*I wear a veil*’.	
**Having a different name** A name might imply one’s origin. ‘*Personally, I believe that foreign names and ages can affect the application. I experienced that*’. ‘*Arabic name is enough to get a no*’.	Some respondents, 2% (*n* = 7), had **changed their name** to a Swedish-sounding name as a strategy for increasing opportunities to be employed. Another strategy: ‘*I erased [name] from my name*’.
**Insufficient language skills** To not speak Swedish good enough was mentioned by 5% as one reason for having one’s competence valued less than the competence of physicians educated in Sweden. ‘*Got several who replied that I need Swedish knowledge even if my email, CV and everything is written in Swedish and they had never talked to me on the phone*’	**Learning the Swedish language** Learning the Swedish language was mentioned by respondents as a facilitating aspect. ‘*I got my jobs because I did not have a strong accent and all of my interviews have gone well due to that*’. Strategies used to learn the language: *Listening to audiobooks *Reading a lot in Swedish *Speaking with Swedish people in general *Using language-training apps *Participating in Swedish language courses *Work Of those who had a position as a physician, 50% perceived that speaking Swedish to Swedes was a very useful aspect that had increased opportunities for finding work as a physician in Sweden. Being married to a Swede was mentioned by one respondent as a facilitating aspect in learning the language, as they speak Swedish at home. The same respondent also mentioned that being married to a Swede had also increased possibilities to integrate into Swedish society. In Sweden, different associations sponsor language cafés where migrants meet and speak, have coffee or tea with the aim of learning the Swedish language. Respondents had not participated in these events to a great extent, and most who had participated did not think of them as valuable for finding work. Swedish language apps and apps with medical terminology and knowledge had not been used to any great extent, and respondents who had used these kinds of apps in general did not perceive them as useful in getting jobs.
**Having the wrong age** It can be assumed that physicians from outside the EU/EEA in general are older than Swedish graduates when they compete for the mandatory medical internship and other positions in the medical field, such as specialty training positions. Of the respondents, 8% perceived that they had been disfavoured related to discrimination based on age. ‘*Due to high age, they do not want to give [mandatory medical internship]*’. Respondents also mentioned that it is more difficult to advance to specific specialties due to being too old. Respondents’ waiting time for the MMI might be related to their age. Respondents with shorter waiting times were slightly younger: 10 months or less of waiting time (*n* = 59): 37 years 11 months or more of waiting time (*n* = 120): 41 years	
**Having the wrong gender** Of the respondents, 2% perceived that they had been disfavoured related to the discrimination based on gender. Of those who had perceived themselves to be disfavoured due to gender, the majority were women (13 of 17). ‘*They asked me about children and how many children I planned*’. In comparison to all respondents, and regarding the mandatory medical internship, men were slightly over-represented in beginning their MMI quicker after having completed route 1 or route 2 (CPP) than women: 10 months or less of waiting time (*n* = 59): 59% men/41% women 11 months or more of waiting time (*n* = 120): 44% men/56% women	
**Lacking work experiences and work references from work in Sweden** Lacking work experience was a perceived reason by 18% of the respondents (*n* = 283) for having one’s competence undervalued during the job-seeking process. ‘*I was told from someone who sits on HR at an employer (which I did not apply for) that job applications from foreign doctors are put in a separate folder which is different from Swedish educated, as they prefer doctors that are experienced in Swedish healthcare*’. ‘*Not familiar with routines, the system*’. ‘*Judgmental employers who will not give foreign physicians without any experience from the Swedish medical labour market*’. ‘*Employers want to hire physicians with experience of Swedish healthcare*’. Lacking work references was a perceived reason by 13% of the respondents (*n* = 283) for having one’s competence undervalued during the job-seeking process.	**Work in the Swedish healthcare and/or medical field** Before the CPP havingworked in the Swedish healthcare and/or medical field as, for example, an assistant nurse, nurse or physician assistant This was in order to: *Learn the language *Learn and/or understand how the routines and system in Swedish healthcare work *Develop contacts *Get into or in touch with the healthcare sector *Demonstrate skills *Develop work references by showing competence during work before the CPP or during CPP internship. *Have many work recommendations from different sites. Regarding work in Swedish healthcare, as physicians in different positions or as other types of healthcare staff, respondents mentioned that they have **struggled**, taking difficult jobs, working hard, sometimes in uncomfortable working hours, and others have been on call at all times, taking jobs with a low salary in a specialty in which they preferred not to work. **Taking a job when over-qualified for it or with a lower salary** Of the respondents, 20% had taken a job beneath their qualifications. ‘*It was quite easy to get a job at a healthcare centre since there is always missing physicians*’. ‘*Took the first job offered and accepted a very low salary in a specialty which I do not like*’. **Doing research** was mentioned by some respondents as an aspect that had increased their opportunities to get a job as a physician in Sweden. However, another MP had left work as a researcher in favour of an under-qualified job in the healthcare sector just to be able to work as a physician later on. **Positive approach** Respondents also mentioned that one needs to be open-minded to new knowledge, be flexible, positive, nice and humble to increase the possibilities to get a job. Also mentioned was that one should adjust to society and not listen to those complaining about the system.
**Lacking contacts within the medical labour market** To lack contacts is a very common barrier in the comments; some examples: ‘*I just sent my CV as I did not know that in Sweden you must have either contacts or recommendations, which I am not used to from my home country*’. ‘*It is difficult to get a job if you do not have any contact in healthcare*’. ‘*I had no contacts with the clinics which I applied for*’. One respondent brought up the topic of nepotism, friendship corruption and bias.	**Having or developing contacts with** *Employers, developed during work before the CPP *Workplaces, developed during the CPP internship *CPP peers, developed during the CPP *Family and relatives *Friends *Peers from the country of origin *Mentors *Having a personal network of Swedes Contacts developed during the CPP were by more than one half of respondents thought to have made it easier for them to get a job as a physician in Sweden: partly, much or to a very much extent (*n* = 236). ‘*Everyone who has contacts gets jobs faster. There are a large group of physicians from the same country [mentions some countries] that help each other a lot and give each other lots of information that others do not get*’. **Strategies to find work without developed contacts** ‘*Sent email to all healthcare centres within [distance] from [city] to receive my first temporary post as a junior doctor*’. ‘*Visited the employer at his workplace as a drop in and then either booked time or had the opportunity to show my qualifications / grades and presented myself as a person, and it has worked well for me and I got a job fast – 3 weeks after my application –but I also could start earlier if I wanted to*’. “*Searched work actively / went to work to ‘mingle’ before the interview*”. ‘*Actively contacted loads of employers in different specialties*’.
**High competition in the labour market regarding the mandatory medical internship, in certain parts of the country (cities) and for advancement within the labour market regarding some specialties** Of the respondents, 70% lived in a big/bigger city in Sweden. Not being able to move due to family was mentioned by some respondents as a barrier to the labour market. For 15% (41, *n* = 269), children living at home affected possibilities or willingness to move; for 29%, children affected to a great extent; and for 19%, partly. For 50% of the respondents, a partner affected the possibility or willingness to move to either a great extent or completely.	**To move or commute** ‘*Aimed at work outside the city, in a rural area*’. ‘*I applied for work everywhere in [region]. I got a call for interviews from some clinics but got my first temporary position far from my home town. Despite young children at home, I accepted it*’. As a strategy to increase job opportunities, 37% of the respondents had applied for a job outside their hometown. ‘*I worked in 3 different towns before I at last got an MMI*’. Of the respondents having or who had had a mandatory medical internship position (*n* = 179), 36% moved for it. Of those who moved (*n* = 66 of 156), 36% were women and 64% were men. **Change specialty** Of the respondents, 19% had changed specialties to increase the opportunities for getting a job.
**Administrative barriers** Respondent mentioned: Lack of/or insufficient *Information about the recertification process *Clinical supervisors during different positions *Medical internships (both before the CPP and the mandatory positions after the CPP) *Cooperation between different authorities (such as the Public Employment Service, migration board, universities and other labour office programmes). *Education positions at CPP The time it takes before being able to work as a physician in Sweden is mentioned by respondents. For example: ‘*If I could have gotten help with the language and a spot at the CPP directly, then I would not have been lost for 5 years*’. Also mentioned is the difference between non-EU/EEA physicians and EU physicians. EU physicians do not have to go through the same process as non-EU/EEA physicians.	**Participating in interventions** Public Employment Service and Public Employment Service office and labour office programme aimed at supporting newly arrived migrants to the labour market was by two-thirds not perceived as having made it easier for them to get a job as a physician in Sweden. The programme had been useful only for a few. A couple of open comments stated that the labour office did not help them, for example: ‘The Public Employment Service’s quick track sounds like a joke but hope this description depends on change since what I experienced in 2013-2014’. *Courses for physicians educated abroad, to facilitate entrance to route 1 or 2. Many participated in different courses, but only a few found them useful or valuable. *Route 2 (CPP) Some mentioned the CPP in general and some as having saved their career. Participating in the CPP is by some seen as a strategy to increase the possibilities to work as a physician in Sweden, as it is also one of the three routes for obtaining the Swedish licence to practice. ‘*The project in itself is very good, that we who do not come from the EU get a place in the Swedish healthcare by updating us how the healthcare system works, and we get to learn the medical terms required to meet a patient*’. However, employers being unfamiliar with the CPP was a perceived reason by 11% of the respondents (*n* = 283) for having one’s competence undervalued during the job-seeking process. *Route 1 Being able to switch from route 2 (the CPP) to route 1, since it is perceived to decrease the time for obtaining the Swedish licence to practise. For some, the change of route meant that they did not need to complete the mandatory medical internship. *Help with CV and job application An MP commented that ‘the right and interesting personal letter [in job application]’ was a facilitating aspect for work. To receive help with CV and personal letter was thought as partly, much or very much to be valuable for increasing job opportunities by almost three-quarters of the respondents. Support *By others going through the same process *Being encouraged by mentor

CPP denotes Complementary Program for Physicians with a medical degree from outside the EU/EEA or Switzerland

*There are seven grounds for discrimination in Sweden: ethnicity, religion or other belief, sex, age, transgender identity or expression, disability and sexual orientation; the last three were not prominent in the data

Experienced barriers also included having one’s competence undervalued during the job-seeking process, which some respondents associated with *originating from another country and/or having a medical degree from abroad*, *insufficient language skills* and *lacking work experience and work references from Sweden*. In some comments, perceptions of having been disfavoured and having competences undervalued both occurred. For example, one respondent applying for the MMI stated:

Very little chance of succeeding despite letters of recommendation [ … ] and good references since I have worked in psychiatry, which is not so much merit for [MMI] and you do not give so much importance to my placements in the home country. Discrimination against foreign doctors and questioning the competences you have on your CV does not help either. One needs to show competence and become overloaded in practice before the employer trusts a foreign doctor, unfortunately. Therefore, foreign workers are most often employed in places where there are major staff shortages.

Of the 16 respondents who had grown up in Sweden or partly resided in Sweden, only two perceived having experiences that could be linked to discrimination, and only one had experienced having their competence undervalued, and the respondents perceived reasons for this were lack of work experience in Swedish healthcare and having been educated abroad (6%, *n* = 16).

### Facilitating aspects

Facilitating aspects and barriers corresponded to each other and are hence presented in the same table (Table 6). For example, lacking contacts was an identified barrier, whereas having contacts was an identified facilitating aspect. In the same table, we also present different strategies used by the MPs for increasing opportunities for employment.

Respondents also had suggestions on what changes would better facilitate entry into the medical labour market: less bureaucracy, living in less segregated areas in which there is a mix of Swedes and foreigners, Swedish language courses, an introduction course on Swedish culture and mentality, more internships, increased CPP and MMI positions, developing a section for MPs within the physicians’ union and employers being more willing to hire foreign physicians.

## Discussion

We explored how MPs with a medical degree from outside EU/EEA after the CPP entered into and advanced within the medical labour market in Sweden as physicians, and we identified perceived barriers and facilitating aspects. We found that even when the MPs had increased their symbolic capital by having a medical education from one country and participating in the CPP in the new country, they still experienced barriers to entering and advancing within the Swedish medical field. These barriers were mainly due to having a medical degree from and/or originating from another country, and they could also be related to the grounds of discrimination and/or having one’s competence undervalued.

Respondents mainly found their first positions via spontaneous job applications, during their CPP internship or via personal contacts. Our results are somewhat in line with previous research showing that MPs are approached with job offers during internships [8]. Respondents’ first job was mainly as a junior doctor, even though the ideal position after the CPP was an MMI. Our results support the indication that it takes longer for MPs to obtain a Swedish medical licence [28]. For our respondents, the mean time to begin the MMI was 16.2 months in comparison to physicians with a medical degree from Sweden or elsewhere in the EU/EEA, who had a mean of 10.3 months of waiting time in 2017 [46]. The waiting time to begin the MMI in Sweden’s largest urban region was 20.4 months for our respondents; for physicians educated in Sweden or elsewhere in the EU/EEA, however, the waiting time was 18.6 months in 2017 [46]. These differences might be due to the difficulties MPs experience, which were explored in our study.

Our results show that the respondents perceived that employers during the job-seeking process undervalued their competence in contrast to that of physicians educated within the country. These results are also in line with research showing that even “where ‘foreign’ qualifications are formally recognized, employers invoke criteria such as the lack of local professional experience” [35]. MPs’ symbolic capital might be formally acknowledged, but in the medical field, it does not seem to attain full recognition. MPs might be perceived as lacking needed ‘national’ capital and perhaps meet national-based protectionism [35], i.e. not being ‘Swedish’ enough. Employers might not know about the CPP or they may mistrust foreign competences, consciously or unconsciously (cf. [35]), just as employers do not always discriminate intentionally [20]. Our results can also be compared to research showing that employed MPs perceive their educations to be undervalued by their peers [22].

Almost one third of the respondents perceived having been disfavoured by employers in their job-seeking process for reasons related to discrimination. Specific combinations, such as age and country of origin, or gender and religion/or other belief, might generate more difficult barriers [47]. Experiences of being disfavoured due to reasons related to discrimination (regulated by law) and/or having one’s competence undervalued (not regulated by law) seem to be intertwined in the creation of barriers to the medical field. Some reasons for not being offered a job might be difficult to grasp or report due to ambiguity. In this respect, other aspects may need to be considered: For instance, is one being discriminated against for not getting a job if that job requires sufficient language skills for patient safety reasons but the job candidate lacks those skills? Having insufficient language skills might be a reason for having one’s competence undervalued and is usually related to being of another ethnicity. In Sweden, ethnicity is considered as a ground for discrimination.

Based on the respondents’ experiences, our interpretation is that the medical field is segmented (cf. [23]) and that MPs as a group are hierarchically positioned lower in the Swedish medical field than physicians trained in the country. However, how they are individually and hierarchically positioned is a complex issue, since MPs are not a homogenous group. Country of origin, country of education or MPs’ beliefs might affect how they are treated in the job application process [17–20]. Also, age and gender may also exert an influence. For example, internationally educated female health professionals appear to struggle more so than men [15, 33]. Individual characteristics influence routes (cf. [33]), and barriers might hence depend on several biographical aspects (cf. [8]).

We found that MPs perceived some aspects as being more facilitative of entering the Swedish medical field and that they had also tried different strategies. Having previously experienced the Swedish healthcare system prior to enrolling in the CPP as an assistant nurse was, for example, perceived as a facilitating aspect, as it helped develop knowledge about the Swedish healthcare field as well as Swedish language skills; these skills can be seen as national-specific and hence “can be converted into ‘national capital’, to legitimize belonging” [35], which might make getting a job easier.

Work in Swedish healthcare previous to enrolling in the CPP and internships during the CPP also seemed to increase opportunities to find work in the future, as the respondents received Swedish work references and developed useful contacts. It was perceived by many respondents that having contacts made it easier to get a job. Having contacts that leads to work can be seen as social capital, which is in turn a form of symbolic capital. Our interpretation is that respondents developed social capital (as in contacts leading to jobs) either strategically or unconsciously in Sweden during work or internships previous to or during the CPP. However, it is also our interpretation that MPs’ possibilities for gaining social capital might be based on first demonstrating their competence in the Swedish healthcare/medical field by, for example, expressing knowledge about the Swedish healthcare system (cf. [35]); such aspects can be perceived as facilitating employment. Having a level of formal education equal to that of physicians educated in Sweden does not seem to be enough, as Salmonsson et al. suggested, because they also ‘need to show that they are able to manage Swedish cultural codes in the health context’ [23]. But we also found that some respondents found their first positions via other MPs participating in the CPP, and hence, CPP peers seemed to be transformed into social capital. Capital other than Swedish national-specific capital can thus be of importance for getting a job, as suggested by Blain et al. [33], who highlighted the support of international medical graduate peers and how both sharing one’s migrant status and medical profession are of importance. The CPP seemed to increase MPs’ networks and opportunities for finding employment, except for facilitating professional development with national-specific capital to fit the new country’s needs and requirements. Research regarding internationally graduated nurses shows that participation in a bridging programme and having a social network seem to be good predictors for securing employment [48], and our results indicate that this might hold true for MPs. Our results are also in line with research showing that MPs after recertification are employed as physicians to a large extent [8].

As our interpretation is that MPs with a medical degree from outside the EU/EEA are, as a group, located hierarchically lower in the Swedish medical field than physicians with a medical education from Sweden, this probably explains why many respondents mentioned struggling to get a job and using different strategies to enter the medical field. Some mentioned working uncomfortable hours, being on call at all times or taking jobs with a low salary in a speciality in which they preferred not to work. Others had moved. MPs also changed specialties or took jobs they were over-qualified for. Those experiencing many difficulties probably also used multiple strategies (cf. [33]). In studies of migrants, it has been shown that one’s name can be used by employers to sort job applications and that name changes can increase opportunities to be hired [20]. In fact, a few respondents in this study did just that, which unfortunately reflects submission to an oppressive norm. On the other hand, if it facilitates an MP’s entrance into the labour market on an individual level, then it is understandable why it would be used as a strategy.

We found that barriers and facilitating aspects were in many cases two sides of the proverbial same coin. In other words, if developing language skills is facilitative, then being unable to speak that language is a barrier. Insufficient language is moreover one of the most common barrier identified in research [8, 19]. We suggest that multiple forms of discrimination can coexist and that in such cases, the struggle to get a job is even harder. On the opposite side however, when MPs develop national-specific capital by, for example, developing language skills *and* increasing their knowledge about the Swedish healthcare field, then job opportunities considerably increase.

### Methodological considerations

First, a limitation with this study was that it only included participants from route 2. However, it is likely that our findings are pertinent to route 1 participants as well. Second, the study was based on self-reported data only, which can be biased; that said, subjective experiences and perceptions offer a preliminary step towards illuminating and addressing barriers and facilitating aspects.

### Implications for practice and future research

By exploring and identifying aspects that influence the transition to a new country’s medical field, interventions can be developed or refined, and policies can be informed. As employers may discriminate unintentionally, they might also undervalue the competence of MPs unconsciously. Therefore, it might be important to train employers to increase their awareness of their own behaviours and prejudices and to realise that even those with good intentions can create or reinforce barriers for MPs. At the societal and individual levels, it is important that MPs participating in bridging programmes find employment afterwards; otherwise, it is a waste of effort, competence and money. In addition, work is an important factor for integration and well-being. For these reasons, the transition between a bridging programme and employment should be as fast as possible.

To integrate language training and MMI in bridging programmes is a further recommendation for practice based on this study. More specified training in the job-seeking process may also be useful, i.e. when to apply, how to write an application and questions that might come up during a job interview. Our study further implies that language training, internship or auscultation opportunities would be useful as soon as possible upon arrival, thus even before bridging programmes.

Future studies could specifically explore route 1 participants with a large enough sample to make valid statistical comparisons regarding the selection of routes within groups of MPs. Also, a corresponding study in which physicians educated in Sweden are contrasted with MPs might elicit alternative perspectives and contribute to knowledge construction.

## Conclusion

Having personal contacts in the medical field who can be used as social capital may increase job opportunities for MPs. An active development of a variety of contacts seems beneficial; this can be established during interventions, such as internships, and with spontaneous job requests or social networks. MPs have very limited opportunities to network in a new country’s medical field during their training; consequently, this study revealed that they are at a disadvantage when entering and advancing within the medical labour market. MPs experience a variety of barriers while attempting to enter and advance within the Swedish medical labour market, partially due to reasons related to discrimination. Many MPs perceived having their competences undervalued due to their origin or education abroad. It is our interpretation that for optimal entry into the labour market, it is vitally important for MPs to get a job or internship as soon as possible within the medical field in order to gain proficiency in field-specific contexts, improve their language skills and develop contacts with colleagues and future employers.

## Data Availability

The dataset generated during and analysed during the current study are not publicly available due to ethical restrictions. The data is protected by confidentiality rules pursuant to the Swedish Public and Privacy Act, which means that no unauthorised person can access the data. This is to protect the participant’s confidentiality and privacy. Any questions regarding the data can be emailed to linda.sturesson@ki.se.

## Abbreviations

CPP: Complementary Program for Physicians with a medical degree from outside EU/EEA and Switzerland
EEA: European Economic Area
EU: European Union
MMI: Mandatory medical internship
MP: Migrant physician
